# Publisher Correction: A meta-analysis of genome-wide association studies identifies multiple longevity genes

**DOI:** 10.1038/s41467-021-22613-2

**Published:** 2021-04-23

**Authors:** Joris Deelen, Daniel S. Evans, Dan E. Arking, Niccolò Tesi, Marianne Nygaard, Xiaomin Liu, Mary K. Wojczynski, Mary L. Biggs, Ashley van der Spek, Gil Atzmon, Erin B. Ware, Chloé Sarnowski, Albert V. Smith, Ilkka Seppälä, Heather J. Cordell, Janina Dose, Najaf Amin, Alice M. Arnold, Kristin L. Ayers, Nir Barzilai, Elizabeth J. Becker, Marian Beekman, Hélène Blanché, Kaare Christensen, Lene Christiansen, Joanna C. Collerton, Sarah Cubaynes, Steven R. Cummings, Karen Davies, Birgit Debrabant, Jean-François Deleuze, Rachel Duncan, Jessica D. Faul, Claudio Franceschi, Pilar Galan, Vilmundur Gudnason, Tamara B. Harris, Martijn Huisman, Mikko A. Hurme, Carol Jagger, Iris Jansen, Marja Jylhä, Mika Kähönen, David Karasik, Sharon L. R. Kardia, Andrew Kingston, Thomas B. L. Kirkwood, Lenore J. Launer, Terho Lehtimäki, Wolfgang Lieb, Leo-Pekka Lyytikäinen, Carmen Martin-Ruiz, Junxia Min, Almut Nebel, Anne B. Newman, Chao Nie, Ellen A. Nohr, Eric S. Orwoll, Thomas T. Perls, Michael A. Province, Bruce M. Psaty, Olli T. Raitakari, Marcel J. T. Reinders, Jean-Marie Robine, Jerome I. Rotter, Paola Sebastiani, Jennifer Smith, Thorkild I. A. Sørensen, Kent D. Taylor, André G. Uitterlinden, Wiesje van der Flier, Sven J. van der Lee, Cornelia M. van Duijn, Diana van Heemst, James W. Vaupel, David Weir, Kenny Ye, Yi Zeng, Wanlin Zheng, Henne Holstege, Douglas P. Kiel, Kathryn L. Lunetta, P. Eline Slagboom, Joanne M. Murabito

**Affiliations:** 1 Max Planck Institute for Biology of Ageing, Cologne, Germany; 2 Molecular Epidemiology, Department of Biomedical Data Sciences, Leiden University Medical Center, Leiden, The Netherlands; 3 California Pacific Medical Center Research Institute, San Francisco, CA USA; 4 McKusick-Nathans Institute of Genetic Medicine, Department of Genetic Medicine, Johns Hopkins University School of Medicine, Baltimore, MD USA; 5 Alzheimer Center Amsterdam, Department of Neurology, Amsterdam Neuroscience, Vrije Universiteit Amsterdam, Amsterdam UMC, Amsterdam, The Netherlands; 6 Department of Clinical Genetics, Amsterdam UMC, Amsterdam, The Netherlands; 7 Delft Bioinformatics Lab, Delft University of Technology, Delft, The Netherlands; 8 The Danish Aging Research Center, Department of Public Health, University of Southern Denmark, Odense C, Denmark; 9 BGI-Shenzhen, Shenzhen, China; 10 China National Genebank, BGI-Shenzhen, Shenzhen, China; 11 Division of Statistical Genomics, Department of Genetics, Washington University School of Medicine, Saint Louis, MO USA; 12 Department of Biostatistics, University of Washington, Seattle, WA USA; 13 Cardiovascular Health Research Unit, Department of Medicine, University of Washington, Seattle, WA USA; 14 Department of Epidemiology, Erasmus MC, Rotterdam, The Netherlands; 15 Department of Biology, Faculty of Natural Science, University of Haifa, Haifa, Israel; 16 Departments of Medicine and Genetics, Albert Einstein College of Medicine, Bronx, NY USA; 17 Institute for Social Research, Survey Research Center, University of Michigan, Ann Arbor, MI USA; 18 Department of Biostatistics, Boston University School of Public Health, Boston, MA USA; 19 School of Public Health, Department of Biostatistics, University of Michigan, Ann Arbor, MI USA; 20 Icelandic Heart Association, Kópavogur, Iceland; 21 Department of Clinical Chemistry, Fimlab Laboratories and Finnish Cardiovascular Research Center—Tampere, Faculty of Medicine and Health Technology, Tampere University, Tampere, Finland; 22 Institute of Genetic Medicine, Newcastle University, Newcastle upon Tyne, UK; 23 Institute of Clinical Molecular Biology, Kiel University, Kiel, Germany; 24 Sema4, a Mount Sinai venture, Stamford, CT USA; 25 Bioinformatics Program, Boston University, Boston, MA USA; 26 Fondation Jean Dausset—CEPH, Paris, France; 27 Clinical Biochemistry and Pharmacology, Odense University Hospital, Odense C, Denmark; 28 Department of Clinical Genetics, Odense University Hospital, Odense C, Denmark; 29 Department of Clinical Immunology, Copenhagen University Hospital, Rigshospitalet, Copenhagen, Denmark; 30 Institute of Health & Society, Newcastle University, Newcastle upon Tyne, UK; 31 MMDN, Univ. Montpellier, EPHE, Unité Inserm 1198, PSL Research University, Montpellier, France; 32 Institute of Neuroscience, Newcastle University, Newcastle upon Tyne, UK; 33 Department of Public Health, University of Southern Denmark, Odense C, Denmark; 34 Centre National de Recherche en Génomique Humaine, CEA-Institut de Biologie François Jacob, Evry, France; 35 Newcastle University Institute for Ageing, Newcastle University, Newcastle upon Tyne, UK; 36 Department of Applied Mathematics and Centre of Bioinformatics, Lobachevsky State University of Nizhny Novgorod, Nizhny Novgorod, Russia; 37 IRCCS Institute of Neurological Sciences of Bologna (ISNB), Bologna, Italy; 38 EREN, UMR U1153 Inserm/U1125 Inra/Cnam/Paris 13, Université Paris 13, CRESS, Bobigny, France; 39 Faculty of Medicine, University of Iceland, Reykjavik, Iceland; 40 Laboratory of Epidemiology and Population Sciences, National Institute on Aging, NIH, Bethesda, MD USA; 41 Department of Epidemiology and Biostatistics, Vrije Universiteit Amsterdam, Amsterdam UMC, Amsterdam, The Netherlands; 42 Amsterdam Public Health Research Institute, Amsterdam, The Netherlands; 43 Department of Microbiology and Immunology, Faculty of Medicine and Health Technology, Tampere University, Tampere, Finland; 44 Department of Complex Trait Genetics, Center for Neurogenomics and Cognitive Research, Vrije Universiteit Amsterdam, Amsterdam, The Netherlands; 45 Faculty of Social Sciences (Health Sciences) and Gerontology Research Center (GEREC), Tampere University, Tampere, Finland; 46 Department of Clinical Physiology, Tampere University Hospital and Finnish Cardiovascular Research Center—Tampere, Faculty of Medicine and Health Technology, Tampere University, Tampere, Finland; 47 Azrieli Faculty of Medicine, Bar Ilan University, Safed, Israel; 48 Hinda and Arthur Marcus Institute for Aging Research, Hebrew SeniorLife, Boston, MA USA; 49 School of Public Health, Epidemiology, University of Michigan, Ann Arbor, MI USA; 50 Institute of Epidemiology and Biobank PopGen, Kiel University, Kiel, Germany; 51 Institute of Translational Medicine, School of Medicine, Zhejiang University, Hangzhou, China; 52 Department of Epidemiology, Graduate School of Public Health, University of Pittsburgh, Pittsburgh, PA USA; 53 Research Unit of Gynecology and Obstetrics, Department of Clinical Research, University of Southern Denmark, Odense C, Denmark; 54 Bone and Mineral Unit, Oregon Health Sciences University, Portland, OR USA; 55 Department of Medicine, Geriatrics Section, Boston Medical Center, Boston University School of Medicine, Boston, MA USA; 56 Department of Epidemiology, University of Washington, Seattle, WA USA; 57 Department of Health Services, University of Washington, Seattle, WA USA; 58 Kaiser Permanente Washington Health Research Institute, Seattle, WA USA; 59 Department of Clinical Physiology and Nuclear Medicine, Turku University Hospital, Turku, Finland; 60 Research Centre of Applied and Preventive Cardiovascular Medicine, University of Turku, Turku, Finland; 61 CERMES3, UMR CNRS 8211—Unité Inserm 988—EHESS—Université Paris Descartes, Paris, France; 62 Institute for Translational Genomics and Population Sciences, Los Angeles Biomedical Research Institute at Harbor-UCLA Medical Center, Torrance, CA USA; 63 Division of Genetic Outcomes, Department of Pediatrics, Harbor-UCLA Medical Center, Torrance, CA USA; 64 Novo Nordisk Foundation Center for Basic Metabolic Research, Section of Metabolic Genetics, and Department of Public Health, Section of Epidemiology, Faculty of Health and Medical Sciences, University of Copenhagen, Copenhagen N, Denmark; 65 MRC Integrative Epidemiology Unit, Bristol University, Bristol, UK; 66 Department of Pediatrics, Harbor-UCLA Medical Center, Torrance, CA USA; 67 Department of Internal Medicine, Erasmus MC, Rotterdam, The Netherlands; 68 Nuffield Department of Population Health, University of Oxford, Oxford, UK; 69 Department of Gerontology and Geriatrics, Leiden University Medical Center, Leiden, The Netherlands; 70 Max Planck Institute for Demographic Research, Rostock, Germany; 71 Department of Epidemiology and Population Health, Albert Einstein College of Medicine, Bronx, NY USA; 72 Center for Healthy Aging and Development Studies, National School of Development and Raissun Institute for Advanced Studies, Peking University, Beijing, China; 73 Center for the Study of Aging and Human Development and Geriatrics Division, Medical School of Duke University, Durham, NC USA; 74 Department of Medicine, Beth Israel Deaconess Medical Center and Harvard Medical School, Boston, MA USA; 75 Broad Institute of MIT & Harvard, Cambridge, MA USA; 76 NHLBI’s and Boston University’s Framingham Heart Study, Framingham, MA USA; 77 Section of General Internal Medicine, Department of Medicine, Boston University School of Medicine, Boston, MA USA

**Keywords:** Genome-wide association studies, Diseases, Ageing, Risk factors

Correction to: *Nature Communications* 10.1038/s41467-019-11558-2, published online 14 August 2019.

The original version of this Article contained an error in Fig. 1c, in which the top SNP was incorrectly labelled ‘rs4293358’. The correct label is ‘rs429358’. This has been corrected in both the PDF and HTML versions of the Article.

