# Supplementary material for: A meta-analysis of genome-wide association studies identifies multiple longevity genes
Source: Nat Commun. 2019 Aug 14;10:3669. doi: 10.1038/s41467-019-11558-2 (PMC6694136; doi:10.1038/s41467-019-11558-2)
Supplement: Supplementary file 1 — Supplementary information [file 41467_2019_11558_MOESM1_ESM.docx]

**A meta-analysis of genome-wide association studies identifies multiple longevity genes.**

Deelen *et al*.

**Table of contents Pages**

1. Supplementary Methods 3-9
2. Supplementary Note 1 10-13
3. Supplementary Figures 14-18
4. Supplementary Tables 19-20
5. Supplementary References 21-23

**Supplementary Methods**

**Study populations**

*100-plus Study/Longitudinal aging study of Amsterdam/Amsterdam dementia cohort*

The 100-plus Study includes Dutch-speaking individuals who (i) can provide official evidence for being aged 100 years or older, (ii) self-report to be cognitively healthy, which is confirmed by a proxy, (iii) consent to donation of a blood sample, (iv) consent to (at least) two home visits from a researcher, and (v) consent to undergo an interview and neuropsychological test battery.^1^ Longitudinal aging study of Amsterdam (LASA) is an ongoing longitudinal study of older adults in the Netherlands initiated in 1991, and represents a sample of 55-85 year-old individuals from the Netherlands.^2, 3^ Amsterdam dementia cohort (ADC) comprises patients who visit the memory clinic of the VU University Medical Center, The Netherlands. The diagnosis of probable AD is based on the clinical criteria formulated by the National Institute of Neurological and Communicative Disorders and Stroke - Alzheimer’s Disease and Related Disorders Association (NINCDS-ADRDA) and based on National Institute of Aging - Alzheimer’s association (NIA-AA).^4, 5^

Genetic variants were determined by standard genotyping or imputation methods. Briefly, we genotyped all individuals using Illumina Global Screening Array (GSAsharedCUSTOM_20018389_A2) and applied established quality control methods. We confirmed high quality genotyping in all individuals (individual call rate >98%, variant call rate >98%) and HWE-departure (d-HWE) was considered significant at p<1x10^-6^. Genotypes were prepared for imputation using provided scripts online (HRC-1000G-check-bim.pl) ^6^. This script compares variant ID, strand and allele frequencies to the HRC reference panel (v1.1, April 2016). Finally, all autosomal variants were submitted to the Michigan imputation server (<https://imputationserver.sph.unimc.edu>)^7^. The server uses SHAPEIT2 (v2.r790) to phase data and imputation to the reference panel (1000G phase 1 version 3) was performed with Minimac3.^7, 8^

The Medical Ethics Committee of the VU University Medical Center (METC) approved the 100-plus Study, LASA and ADC. All participants and/or their legal guardians gave written informed consent for participation in clinical and genetic studies.

*Age/Gene Environment Susceptibility Study*

The Reykjavik Study cohort originally comprised a random sample of 30,795 men and women born in 1907-1935 and living in Reykjavik in 1967. A total of 19,381 attended, resulting in a 71% recruitment rate. The study sample was divided into six groups by birth year and birth date within month. One group was designated for longitudinal follow-up and was examined in all stages. One group was designated a control group and was not included in examinations until 1991. Other groups were invited to participate in specific stages of the study. Between 2002 and 2006, the AGES-Reykjavik study re-examined 5,764 survivors of the original cohort who had participated earlier in the Reykjavik Study. The AGES/Reykjavik Study GWAS was approved by the National Bioethics Committee (VSN: 00‐063) and the Data Protection Authority.

DNA from participants was genotyped using the Illumina 370CNV BeadChip array on 3,664 participants. Samples were excluded from the dataset based on sample failure, genotype mismatch with reference panel, and sex mismatch, resulting in clean genotype data on 3,219 individuals. Standard protocols for working with Illumina data were followed, with clustering score greater than 0.4. Prior to genotype imputation, single nucleotide polymorphisms (SNPs) were excluded using filters based on call rate (<97%), Hardy-Weinberg Equilibrium (HWE) (P <1.0 x 10^-6^), mishap (P <1.0 x 10^-9^), and mismatched positions between Illumina, dbSNP and/or HapMap resulting in 325,094 SNPs passing all QC (of 353,202 prior to cleaning steps). Imputation was conducted against 1000 Genomes project reference panel (version Phase 1 integrated release v3, March 2012).

*CEPH centenarian cohort*

French centenarians were recruited when they were in their 100th year or beyond. French siblings were recruited when at least two siblings fulfilling age criteria of 90 years or older were alive in a family. The mean age of centenarians and unrelated siblings was respectively 104 and 100 years (age at death or age at last contact). In total 1,234 elderly were included in the study (998 centenarians and 236 unrelated members from a sibling). DNA was extracted from peripheral blood lymphocytes by standard methods.^9^

*Cardiovascular Health Study*

The Cardiovascular Health Study (CHS) is a population-based cohort study of risk factors for coronary heart disease and stroke in adults ≥65 years conducted across four field centers.^10^ The original predominantly European ancestry cohort of 5,201 persons was recruited in 1989-1990 from random samples of the Medicare eligibility lists; subsequently, an additional predominantly African-American cohort of 687 persons was enrolled for a total sample of 5,888. Blood samples were drawn from all participants at their baseline examination and DNA was subsequently extracted from available samples. Genotyping was performed at the General Clinical Research Center’s Phenotyping/Genotyping Laboratory at Cedars-Sinai among CHS participants who consented to genetic testing and had DNA available using the Illumina 370CNV BeadChip system (for European ancestry participants, in 2007) or the Illumina HumanOmni1-Quad_v1 BeadChip system (for African-American participants, in 2010). CHS was approved by institutional review committees at each field center and individuals in the present analysis had available DNA and gave informed consent including consent to use of genetic information for the study of cardiovascular disease.

European ancestry participants were excluded from the GWAS study sample due to the presence at study baseline of coronary heart disease, congestive heart failure, peripheral vascular disease, valvular heart disease, stroke or transient ischemic attack or lack of available DNA. Beyond laboratory genotyping failures, participants were excluded if they had a call rate<=95% or if their genotype was discordant with known sex or prior genotyping (to identify possible sample swaps). After quality control, genotyping was successful for 3,268 European ancestry and 823 African-American participants.

*Cardiovascular Risk in Young Finns Study*

Living controls for the Vitality 90+ study were derived from the Cardiovascular Risk in Young Finns Study (YFS). The YFS cohort is a Finnish longitudinal population study sample on the evolution of cardiovascular risk factors from childhood to adulthood. For the present analysis, DNA was collected at the follow-up examination in 2011 when the participants were aged 34 to 49 years. Genotyping was successful for 1,995 participants. The study and data collection protocols have been described in detail previously.^11^ YFS was approved by the Ethics Committee of the Hospital District of Southwest Finland. All participants gave their written informed consent.

*Chinese Longitudinal Healthy Longevity Survey*

The Chinese Longitudinal Healthy Longevity Study (CLHLS) is a large-scale population-based study on health status and quality of life of the elderly in 23 provinces (out of 31 provinces) of China since 1998 with 8 waves so far. The study covers approximately 85% of the total population of China and was conducted to shed light on the determinants of human healthy longevity. The CLHLS tried to interview all consenting centenarians in the sampled counties and cities. For each centenarian interviewee, compatible nearby un-related elderly and younger participants were interviewed, including one middle-aged adult aged 40-64, 1.5 young-old adult aged 65-79, one octogenarian aged 80-89 and one nonagenarian aged 90-99. “Nearby” is loosely defined – it could mean in the same village or the same street if available, or in the same town or in the same sampled county or city.^12^ In the CLHLS GWAS, we use centenarians as cases and middle-aged adults as controls; all of the DNA samples from middle-age controls were collected in the same county/city as the nearby centenarians.^13^

Phenotype data were collected in the CLHLS using internationally standardized questionnaires adapted to the Chinese cultural and social context. Extensive evaluations of the data quality of the CLHLS, including assessments of mortality rate, proxy use, non-response rate, sample attrition, reliability and validity of major health measures, and the rates of logically inconsistent answers, have shown that the data from the CLHLS surveys are of reasonably good quality.^14, 15^

In the CLHLS GWAS, 2,578 centenarians and 2,387 middle-age controls were genotyped using the Illumina HumanOmniZhongHua-8 BeadChips (900K SNPs). For the sample filtering, 339 individuals for whom the genotypes were generated with a call rate less than 95% were excluded. The 134 individuals who had identity-by-state probabilities with PI_HAT > 0.25 were excluded based on the IBD analysis implemented in PLINK. The 15 outliers in the principle components analysis (PCA) analysis were also excluded. Finally, after various stages of sample filtering, 2,178 centenarians (mean age 102.7 ± 3.49 (SD)) and 2,299 middle-age controls (mean age 48.4 ± 7.44 (SD)) were included in the CLHLS GWAS dataset. We also conducted quality-control filtering of the GWAS data from the total 4,477 individuals. SNPs with call rates of less than 90% were removed from our GWAS dataset; SNPs were also excluded if they had a MAF less than 1% or if there was significant deviation from Hardy-Weinberg equilibrium in the samples, defined as P <1 x 10^−5^. To further increase genome coverage, we performed imputation analysis to infer the genotypes of all SNPs (MAF ≥ 0.01) using Impute2 software and the 1000 Genomes Project integrated phase 1 release as reference panel. SNPs with a quality score (Rsq) of < 0.9 were discarded before analysis. After standard GWAS quality-control filtering for subjects and SNPs as described above, we obtained data for 5.6 million SNPs in 2,178 centenarian cases and 2,299 middle-age controls for the subsequent GWAS analyses. The Research Ethics Committees of Peking University and Duke University granted approval for the Protection of Human Subjects for the CLHLS, including collections of DNA sample used for CLHLS GWAS. The survey respondents who contributed their DNA samples gave informed consent before participation.

*Danish longevity study*

The Danish Longevity Study (DKLS) consists of participants drawn from seven nation-wide surveys collected at the University of Southern Denmark; the Study of Danish Old Sibs (DOS), the 1905 Birth Cohort Study, the 1910 Birth Cohort Study, the 1911-12 Birth Cohort Study, the 1915 Birth Cohort Study, the Longitudinal Study of Danish Centenarians (LSDC), and the Longitudinal Study of Ageing Danish Twins (LSADT). Briefly, DOS was initiated in 2004 and includes families in which at least two siblings were ≥ 90 years of age at intake. The LSDC and 1905, 1910, and 1915 Birth Cohort Studies are prospective follow-up studies initiated in 1995, 1998, 2010, and 2010, when participants were 100, 92-93, 100, and 95 years of age, respectively.^16^ The 1911-1912 cohort study consists of individuals reaching the age of 100 years in the period from May 2011 to July 2012,^17^ and LSADT was initiated in 1995 and includes Danish twins ≥ 70 years of age.^18^ From DOS and LSADT, one individual from each sib-ship or twin pair was randomly selected among participants that had reached an age of at least 91 years for DOS, and 90 years for LSADT. From the 1905 and 1915 Birth Cohort Studies, participants were selected among individuals reaching an age of minimum 96 years. In total, the DKLS includes 1,003 individuals (244 (24.3%) men) with a mean age at intake of 97.4 years (range 90-102.5 years). Of these, 960 lived to an age greater than or equal to the age at the 90^th^ survival percentile specific to country, sex, and birth cohort and 610 lived to an age greater than or equal to the age at the 99^th^ survival percentile.

DNA was extracted from whole blood using standard methods,^19^ or from filter cards using the Extract-N-Amp Blood PCR Kit (Sigma Aldrich, St. Louis, MO, USA) followed by amplification using the GenomePlex Complete Whole Genome Amplification (WGA) Kit (Sigma Aldrich, St. Louis, MO, USA).

Signed informed consents were obtained from all participants. Collection and use of biological material and survey information were approved by the Regional Scientific Ethical Committees for Southern Denmark, and the study was approved by the Danish Data Protection Agency.

*Danish longevity study II*

The Danish longevity study II (DKLSII) consisted of 944 long-lived individuals (261 (27.7%) men) with a mean age at intake of 95.3 years (age range 94.7-95.9 years) drawn from the 1915 Birth Cohort Study and 772 younger controls (304 (39.4%) men) with a mean age at intake of 63.1 years (age range 56-71 years) drawn from the study of Middle-Aged Danish Twins (MADT). Briefly, the 1915 Birth Cohort study is a nation-wide study of individuals born in 1915. The study was initiated in 2010, when participants were around 95 years of age.^16^ MADT was initiated in 1998 and includes twins randomly chosen from the birth years 1931-1952.^20^ The individuals from MADT included here were all born from 1940-1952 and only one twin from each twin pair was included. Of the 944 long-lived individuals, all lived to an age greater than or equal to the age at the 90^th^ survival percentile specific to country, sex, and birth cohort and 298 lived to an age greater than or equal to the age at the 99^th^ survival percentile (as per June 30th 2017).

DNA was extracted from dried blood spot cards using either the DNA Mini or Micro Kits (Qiagen, Hilden, Germany) or the Extract-N-AmpTM Blood PCR Kit (Sigma Aldrich, St. Louis, MO, USA), or from whole blood using a manual or a semi-automatic (Autopure, Qiagen, Hilden, Germany) salting out method.^19^ *APOE* alleles were assessed from the rs7412 and rs429358 genotypes produced using predesigned Taqman ® SNP Genotyping Assays (Life Technologies, Carlsbad, CA, USA) following the manufacturer’s instructions.

Signed informed consents were obtained from all participants. Collection and use of biological material and survey information were approved by the Regional Scientific Ethical Committees for Southern Denmark, and the study was approved by the Danish Data Protection Agency.

*Framingham Heart Study*

The Framingham Heart Study (FHS) is a three-generation, single-site, community-based, prospective cohort study that was initiated in 1948 to investigate risk factors for cardiovascular disease including stroke. The population of Framingham was primarily whites in 1948. The FHS comprises three generations of participants: the original cohort followed since 1948 (Original or Gen1);^21^ their offspring and spouses of the offspring, followed since 1971 (Offspring or Gen2);^22^ and children from the largest offspring families enrolled in 2002 (Gen3).^23^ The Original cohort enrolled 5,209 men and women who comprised two-thirds of the adult population then residing in Framingham, MA, USA. Survivors continue under active surveillance. The Offspring cohort comprises 5,124 persons (including 3,514 biological offspring) who have been examined approximately once every 4 to 8 years. The first examination of the Gen3 was completed in July 2005 and involved 4,095 participants. At that time Offspring spouses not previously enrolled who were a biological parent of a Gen 3 participant underwent recruitment to the New Offspring Spouse cohort to complete family pedigrees. All cohorts continue under active surveillance for cardiovascular events. The FHS also follow two multi-ethnic cohorts, Omni group 1 and Omni group 2 to reflect the diversity of the town of Framingham, MA at present time. Participants in the Omni cohorts are not included in this analysis due to the small number of participants achieving the case definition.

In the 1990s and early 2000s, DNA samples were collected in the Original, Offspring, Third Generation, and Offspring Spouse cohorts of the FHS to establish an invaluable resource for genetic research. All individuals provided consent for genotyping. In 2007, the FHS entered a new phase with the conduct of genotyping for the NHLBI funded SNP-Health Association Resource (SHARe) project using approximately 550,000 SNPs (Affymetrix 500K mapping array plus Affymetrix 50K supplemental array) in over 9,300 subjects from the three generations of subjects (including over 1,500 families). Individuals that did not pass QC criteria (call rate < 97%, extreme heterozygosity or high Mendelian error rate) were excluded. After QC, a total of 8,481 genotyped individuals were available for imputations. Imputations were performed with miniMACH3 using the HRC release 1 reference panel for SNPs passing the following criteria: call rate ≥ 97%, pHWE ≥ 10^-6^, < 100 Mendel errors, and MAF ≥ 1%.

*Genetics of Healthy Aging Study*

The individuals investigated in this study participate in Genetics of Healthy Aging (GEHA) Study.^24^ Families participating in the GEHA study have at least two siblings meeting four inclusion criteria: (i) participants are at least 90 years old, (ii) participants have at least one living brother or sister who fulfils the first criterion and is willing to participate, (iii) the nonagenarian sibship has an identical mother and father, and (iv) the parents of the nonagenarian sibship are European and Caucasian. In total, 2249 sibships have been recruited. From each pair the eldest siblings from the Netherlands, Denmark, France, and Bologna, and GEHA controls from France and Bologna were genotyped. In accordance with the Declaration of Helsinki, written informed consent was obtained from all participants prior to entering the study. Good clinical practice guidelines were maintained. The study protocol was approved by the local medical ethical committees of the 11 participating countries before the start of the study. In this study, analyses were performed separately for each country.

*Genomics of extremely Overweight Young Adults*

Controls for the Danish Longevity Study and for the Danish GEHA samples were derived from the Danish genome-wide association study GOYA (Genomics of extremely Overweight Young Adults).^25^ In this study, female controls initially came from the Danish National Birth Cohort (1996-2002) where they were drawn as a random cohort sample from 67,863 women who gave birth to a live born infant and provided a blood sample during pregnancy. Genotyping was successful in 2,021 women. Similarly, the male controls were a random cohort sample, initially based on one in every hundred of 362,000 Caucasian men, drafted in Copenhagen from 1943-77 (n = 3,601). In 1992-94, half of the random cohort still living in the Copenhagen area were invited to a follow-up where blood samples were taken. Genotyping was successful in 796 men.

*German longevity study*

In total, 1,613 individuals of 95 years and older were recruited (mean age: 98.89 years; age range: 95-110 years; 72.97 % females); among these, 748 individuals were 100 years and older (mean age: 101.3 years, age range: 100-110 years; 80.48 % females). The 1,613 individuals all belonged to the 1% oldest individuals (>99th percentile) in Germany considering the respective dates of birth. The 4,215 unrelated younger controls were 18 to 75 years old (mean age: 50.13 years; all < 60^th^ percentile; 57.44 % females). The German samples were partly collected with the help of the PopGen biobank.^26, 27^ Written informed consent was obtained from all individuals. The project was approved by the Ethics Committee of the Medical Faculty of Kiel University. Genotyping was performed by TaqMan (Thermo Fisher Scientific Inc., Waltham, USA) on a 7900HT Fast Real-time PCR System (Thermo Fisher Scientific Inc., Waltham, USA). Data analysis was performed using PLINK 1.9.^28^

*Health and Retirement Study*

The Health and Retirement Study (HRS) is a longitudinal survey of a representative sample of Americans over the age of 50.^29^ The current sample is over 26,000 persons in 17,000 households. Respondents are interviewed every two years about income and wealth, health and use of health services, work and retirement, and family connections. DNA was extracted from saliva collected during a face-to-face interview in the respondents' homes. These data represent respondents who provided DNA samples and signed consent forms in 2006, 2008, and 2010. Respondents were removed if they had missing genotype or phenotype data. The final European American sample includes all self-reported non-Hispanic whites that had PC loadings within ±one standard deviations of the mean for eigenvectors 1 and 2 in the PC analysis of all unrelated study subjects. The final African American sample includes all self-reported African Americans within two standard deviations of the mean of all self-identified African Americans for eigenvector 1 and ± one standard deviation of the mean for eigenvector 2 in the PC analysis of all unrelated study subjects.

*Long Life Family Study*

The Long Life Family Study (LLFS) is a family-based study of healthy aging and longevity that recruited 539 families and 4,953 family members selected for familial longevity.^30, 31^ Participants were enrolled at three American field centers (Boston, Pittsburgh and New York), and a European field center in Denmark. Potential probands were recruited based on older age, capacity to understand the study and their Family Longevity Selection Score (FLoSS). The FLoSS quantifies the degree of familial longevity using sex and birth-year cohort survival probabilities of the proband and their siblings.^30^ Eligibility of sibships for the study was based on a FLoSS score >7 and having at least one living sibling and at least one offspring willing to be enrolled in the study. Socio-demographic, medical history data, current medical conditions and medications, physical and cognitive function data, and blood samples were collected via in-person visits and phone questionnaires for all subjects at the time of enrollment as described elsewhere, and patients are followed-up annually to track vital and health status. The age of the oldest participants was validated.^32^ Genome-wide genotype data were generated using Illumina SNP arrays,^33^ and genome-wide genotype data are available from dbGaP (dbGaP Study Accession: phs000397.v1.p1). All subjects provided informed consent approved by the field centers IRB.

*Leiden Longevity Study*

The Leiden Longevity Study (LLS) consists of 421 long-lived families of European descent. Families were included if at least two long-lived siblings were alive and fulfilled the age criterion of 89 years or older for males and 91 years or older for females, representing <0.5% of the Dutch population in 2001.^34^ In total, 944 long-lived proband siblings (mean age=94 years, range=89–104), 1,671 offspring (mean age=61 years, range=39–81) and 744 spouses thereof (mean age=60 years, range=36–79) were included. In the LLS, the spouses are considered controls. DNA from the LLS was extracted from white blood cells at baseline using conventional methods and genotyping was performed with Illumina Human660W-Quad and OmniExpress BeadChips (Illumina, San Diego, CA, USA).^35, 36^ In this study, we only used the nonagenarians (as cases) and controls for which the identity was confirmed after genotyping.

*Longevity Gene Project*

This is a study on Ashkenazi Jewish adults of ages 65 and older that were recruited to explore why some people enjoy extremely long life spans (survived to at least age 95 years old) with good physical health and dementia free at the time of enrollment.^37^ Siblings, offspring (having at least one parent who lived to age 95) and spouses of offspring were also enrolled and additional unrelated population controls (neither parent survived to age 95) were selected based on lack of familial longevity.^38^ Genotyping was done in a single center (CIDR) using HumanOmniExpress (Illumina, San Diego, CA, USA) array, and was imputed to the 1000 genomes.^39^

*Osteoporotic Fractures in Men Study*

The Osteoporotic Fractures in Men Study (MrOS) is a multi-center prospective, longitudinal, observational study of risk factors for vertebral and all non-vertebral fractures in older men, and of the sequelae of fractures in men. The original specific aims of the study include: (1) to define the skeletal determinants of fracture risk in older men, (2) to define lifestyle and medical factors related to fracture risk, (3) to establish the contribution of fall frequency to fracture risk in older men, (4) to determine to what extent androgen and estrogen concentrations influence fracture risk, (5) to examine the effects of fractures on quality of life, (6) to identify sex differences in the predictors and outcomes of fracture, (7) to collect and store serum, urine and DNA for future analyses as directed by emerging evidence in the fields of aging and skeletal health, and (8) define the extent to which bone mass/fracture risk and prostate diseases are linked.

The MrOS study population consists of 5,994 community dwelling, ambulatory men aged 65 years or older from six communities in the United States (Birmingham, AL; Minneapolis, MN; Palo Alto, CA; Monongahela Valley near Pittsburgh, PA; Portland, OR; and San Diego, CA). Inclusion criteria were designed to provide a study cohort that is representative of the broad population of older men. The inclusion criteria were: (1) ability to walk without the assistance of another, (2) absence of bilateral hip replacements, (3) ability to provide self-reported data, (4) residence near a clinical site for the duration of the study, (5) absence of a medical condition that (in the judgment of the investigator) would result in imminent death, and (6) ability to understand and sign an informed consent. To qualify as an enrollee, the participant had to provide written informed consent, complete the self-administered questionnaire (SAQ), attend the clinic visit, and complete at least the anthropometric, DEXA, and vertebral X-ray procedures.

Genomic DNA from participants in the MrOS Study was extracted from whole blood samples collected at the baseline visit using the Flexigene protocol (Qiagen, Valencia, CA, USA) at the University of Pittsburgh. Among the 5994 MrOS participants enrolled at the baseline visit, 5530 participants provided blood samples, had DNA extracted, and consented to genetic testing. DNA samples from these 5530 participants were submitted to the Broad Institute for whole-genome genotyping. At the Broad Institute, DNA concentration was assayed by Picogreen and DNA solution volume was measured. Among the 5530 MrOS DNA samples, 5485 had sufficient DNA quantity and DNA solution volume and underwent whole-genome genotyping. All DNA samples eligible for whole-genome genotyping were genotyped using Sequenom iPLEX technology for a 24-SNP “fingerprint” panel. The Illumina HumanOmni1_Quad_v1-0 H genotyping array, containing 1,140,419 probes, was used for whole-genome genotyping. Samples were randomized to 96-well genotyping plates by sex and clinic site. Pairwise concordance between 81 duplicate samples was 100%. 119 replicates of samples from HapMap trios of CEU and YRI populations and singletons from CHB and JPT populations were also genotyped and compared to published HapMap genotypes. Concordance was 99.7% for CEU and YRI samples and was 95.0-99.7% for CHB and JPT samples.

Genotypes were called using a clustering algorithm in Illumina’s BeadStudio software at the Broad Institute. All genotype quality control was performed using custom scripts generated in the R statistical Language (version 2.12 and using the packages netCDF, v. 4.11 and GWASTools). SNPs were excluded with GenTrain scores <0.6, cluster separation scores <0.4, missing rate > 2.5%, > 1 discordant call between duplicate samples, autosomal HWE P-value < 10^-6^, or MAF < 0.01. In addition, genotype clusters for SNPs on chrX, chrY, chrXY and chrMT were reviewed manually. Heterozygous X-chromosome genotypes in MrOS were set to missing. For MrOS samples, 739,528 SNPs passed QC.

Samples with call rates < 97% were excluded. Additional samples were excluded based on: (1) genotypic sex mismatch using X and Y chromosome probe intensities, (2) relatedness among genotyped samples using the kinship coefficient that estimates probability that alleles are identical-by-descent, and (3) ethnic outliers. Principal component analysis (PCA) was performed to detect evidence for population structure, exclude genetic ancestry outliers, and produce principal components to include in regression models to adjust for genetic ancestry. Unrelated samples from the CEU, YRI, CHB, and JPT HapMap populations were used to generate principal components. Eigenvectors were calculated after removal of samples determined to be twins, sample with multiple cousins, and replicate samples as determined by IBD analysis. SNPs used for the calculation of the final eigenvectors had MAF>0.001 and linkage disequilibrium values < 0.05. For MrOS, eigenvectors were calculated using all ethnicities to identify non-European ancestry samples, and again using those samples within 4SD of the mean after excluding non-European ancestry samples.

In MrOS, 739,528 SNPs in 4615 samples of European ancestry that passed QC were used for genotype imputation. Prior to imputation, scripts developed by Will Rayner (<http://www.well.ox.ac.uk/~wrayner/tools>) were used to compare MrOS genotype data with genotype data from 1000 Genomes Phase 3 EUR samples. Strand on which alleles were represented were updated to match the reference panel. SNPs were removed if they were coded with A/T or G/C alleles with MAF>0.4, SNP alleles differed from the reference panel, SNPs were absent from reference panel, or if the allele frequency differed by more than 0.2 from the EUR samples in the reference panel.

Genotype imputation was performed using the University of Michigan imputation server. Sample genotypes were phased using ShapeIt2, and imputation was performed using minimac3. Reference panel used was 1000 Genomes Phase 1 version 3. Genotype dosage was extracted from VCF files using DosageConvertor to produce PLINK dosage files (https://genome.sph.umich.edu/wiki/DosageConvertor).

*Newcastle 85+ Study*

The longitudinal Newcastle 85+ Study recruited in 2006-7 a total cohort of 1042 individuals all born in 1921, of whom 852 underwent full assessment (the remainder agreeing to review of medical records).^40^ Given the near universal coverage of the NHS among people of this age and in this region, effectively all those in the age group were approached to participate, including those in care homes and with disabilities and/or cognitive impairment. A good level of recruitment was achieved with a high degree of representativeness in the study sample. DNA was extracted at baseline using conventional methods for 765 individuals for whom adequate blood samples were available. Genotype data were obtained for 710 individuals and, following quality control checks, 642 individuals were retained. Limiting inclusion to individuals with survival greater than or equal to the 90^th^ percentile (males reaching the age of 90 and females reaching the age of 94) resulted in a total of 134 males and 81 females contributing to the current analysis.

*Rotterdam study*

The Rotterdam Study (RS) is a prospective population-based study designed to investigate the determinants of disease occurrence and progression in the elderly.^41^ The RS cohort was initially defined in 1990 among 7983 persons of 55 years of age or over (RS-I) living in the well-defined Ommoord district in Rotterdam, The Netherlands. All participants underwent an interview at home and extensive physical examination at baseline and during follow-up examinations occurring every 3–4 years. The cohort was further extended in 2000 (RS-II) and 2005 (RS-III), establishing a total of 14,926 participants. In the current study, participants of RS-I were included and follow-up information until March 2015 was used. The Rotterdam Study has been approved by the Medical Ethics Committee of the Erasmus MC (registration number MEC 02.1015) and by the Dutch Ministry of Health, Welfare and Sport (Population Screening Act WBO, license number 1071272-159521-PG). The Rotterdam Study has been entered into the Netherlands National Trial Register (NTR; www.trialregister.nl) and into the WHO International Clinical Trials Registry Platform (ICTRP; www.who.int/ictrp/network/primary/en/) under shared catalogue number NTR6831. All participants provided written informed consent to participate in the study and to have their information obtained from treating physicians.

*Study of Osteoporotic Fracture*

The Study of Osteoporotic Fractures (SOF) is a prospective multicenter study of risk factors for vertebral and non-vertebral fractures. The cohort is comprised of 9704 community dwelling women 65 years old or older recruited from populations-based listings in four U.S. areas: Baltimore, Maryland; Minneapolis, Minnesota; Portland, Oregon; and the Monongahela Valley, Pennsylvania. Women enrolled in the study were 99% Caucasian with African American women initially excluded from the study due to their low incidence of hip fractures. A cohort of AA women was recruited at the 6^th^ Visit.

The inclusion criteria were: 1) 65 years or older, (2) ability to walk without the assistance of another, (3) absence of bilateral hip replacements, (4) ability to provide self-reported data, (5) residence near a clinical site for the duration of the study, (6) absence of a medical condition that (in the judgment of the investigator) would result in imminent death, and (7) ability to understand and sign an informed consent. To qualify as an enrollee, the participant had to provide written informed consent, complete the self-administered questionnaire (SAQ), attend the clinic visit, and complete at least the anthropometric measures. The SOF study recruited only women.

In collaboration with Roche Molecular Systems (Alameda, CA), DNA from participants of the Study of Osteoporotic Fractures (SOF) was extracted from either buffy coat or whole blood samples collected at either visit 2 (1989–1990) or visit 6 (1997–1998). Among the 9704 SOF participants enrolled at the baseline visit, 6795 participants provided blood samples and consented to genetic testing. Among these 6795 SOF participants, DNA samples from 4117 participants had sufficient DNA quantity and were submitted to the Broad Institute for whole-genome genotyping. At the Broad Institute, DNA concentration was assayed by Picogreen and DNA solution volume was measured. Among the 4117 SOF DNA samples, 3924 had sufficient DNA quantity and DNA solution volume and underwent whole-genome genotyping. All DNA samples eligible for whole-genome genotyping were genotyped using Sequenom iPLEX technology for a 24-SNP “fingerprint” panel.

Genotyping and QC as performed for MrOS were also applied to SOF samples with the following exceptions: heterozygous genotypes on the X were not set to missing, In SOF, 739,528 SNPs in 3625 samples of European ancestry that passed QC were used for genotype imputation. Pre-imputation QC using scripts from Will Rayner, genotype imputation, post-imputation data formatting, and association analysis was performed in the same manner as for the MrOS samples.

*Supplementation in Vitamins and Mineral Antioxidants*

Controls for CEPH Centenarians were selected in a population-based sample of French subjects that had participated in the Supplementation in Vitamins and Mineral Antioxidants (SU.VI.MAX) study.^42^

*Vitality 90+ project*

The cases were drawn from the Vitality 90+ project, which investigates the health and social situation of a population aged 90 and older as previously described.^43^ The study population used in the present study was based on the cohort consisted of all nonagenarians born in 1909/1910 and living in Tampere municipality in southern Finland (total population approximately 210,000). Of the cohort, 20% were men and 80% women, and 72% were still living at home. Of the 537 persons born in those years, 285 volunteered for the study. During home visits, participants were interviewed and questionnaires filled with the help of a study nurse. After informed consent, blood samples were drawn from 285 individuals aged 89 to 91 years. Of these nonagenarians, DNA was available and genotyping successful for 254 participants. Of the 254 genotyped individuals, 226 were aged over the country-, birth year cohort-, and sex-specific 90^th^ percentile survival probability at baseline or during 4 years of follow-up and were therefore included in the present analysis. The ethical committee of the city of Tampere approved the study plan.

*Wellcome Trust Case Control Consortium 2*

UK population-based control data were obtained from the Wellcome Trust Case Control Consortium 2 (WTCCC2) (https://www.wtccc.org.uk/ccc2) and comprise individuals from two collections, the 1958 Birth Cohort (58C) and a National Blood Donor Collection (NBS). In the current study we used all individuals passing genotype quality control checks (n=5159) as described in.^44^

**Supplementary Note 1**

**Acknowledgements**

*100-plus Study/Longitudinal aging study of Amsterdam/Amsterdam dementia cohort*

Research at the VUmc Alzheimer center is part of the neurodegeneration research program of Amsterdam Neuroscience ([www.amsterdamresearch.org](http://www.amsterdamresearch.org)). The VUmc Alzheimer Center is supported by Stichting Alzheimer Nederland (WE09.2014-03) and Stichting VUmc fonds. The clinical database structure was developed with funding from Stichting Dioraphte (VSM 14 04 14 02). The Dutch case-control study is part of EADB (European Alzheimer DNA biobank) funded by JPcofundNL (ZonMW project number: 733051061). This work was in part carried out on the Dutch national e-infrastructure with the support of SURF Cooperative.

*Age/Gene Environment Susceptibility Study*

This work was supported by National Institutes of Health (contracts N01-AG-12100 and HHSN271201200022C); the National Institute on Aging Intramural Research Program; Hjartavernd (the Icelandic Heart Association); the Althingi (the Icelandic Parliament).

*CEPH centenarian cohort*

The CEPH centenarian cohort study was funded by the ‘‘Ministère de l’Enseignement supérieur et de la Recherche’’ and the Commissariat à L'Energie Atomique-Centre National de Génotypage. The authors thank the CEPH Biological Resource Centre and the CNRGH Genotyping lab for technical assistance.

*Cardiovascular Health Study*

This CHS research was supported by NHLBI contracts HHSN268201200036C, HHSN268200800007C, HHSN268201800001C, N01HC55222, N01HC85079, N01HC85080, N01HC85081, N01HC85082, N01HC85083, N01HC85086; and NHLBI grants U01HL080295, R01HL087652, R01HL105756, R01HL103612, R01HL120393, R01HL085251, and U01HL130114 with additional contribution from the National Institute of Neurological Disorders and Stroke (NINDS). Additional support was provided through R01AG023629 from the National Institute on Aging (NIA). A full list of principal CHS investigators and institutions can be found at [CHS-NHLBI.org](http://chs-nhlbi.org/). The provision of genotyping data was supported in part by the National Center for Advancing Translational Sciences, CTSI grant UL1-TR-001881, and the National Institute of Diabetes and Digestive and Kidney Disease Diabetes Research Center (DRC) grant DK063491 to the Southern California Diabetes Endocrinology Research Center. The content is solely the responsibility of the authors and does not necessarily represent the official views of the National Institutes of Health.

*Cardiovascular Risk in Young Finns Study*

The Young Finns Study has been financially supported by the Academy of Finland: grants 286284, 134309 (Eye), 126925, 121584, 124282, 129378 (Salve), 117787 (Gendi), and 41071 (Skidi); the Social Insurance Institution of Finland; Competitive State Research Financing of the Expert Responsibility area of Kuopio, Tampere and Turku University Hospitals (grant X51001); Juho Vainio Foundation; Paavo Nurmi Foundation; Finnish Foundation for Cardiovascular Research ; Finnish Cultural Foundation; The Sigrid Juselius Foundation; Tampere Tuberculosis Foundation; Emil Aaltonen Foundation; Yrjö Jahnsson Foundation; Signe and Ane Gyllenberg Foundation; Diabetes Research Foundation of Finnish Diabetes Association; and EU Horizon 2020 (grant 755320 for TAXINOMISIS); and European Research Council (grant 742927 for MULTIEPIGEN project). We thank the teams that collected data at all measurement time points; the persons who participated as both children and adults in these longitudinal studies; and biostatisticians Irina Lisinen, Johanna Ikonen, Noora Kartiosuo, Ville Aalto, and Jarno Kankaanranta for data management and statistical advice.

*Chinese Longitudinal Healthy Longevity Survey*

The relevant research of the CLHLS GWAS for present study is supported by the National Natural Science Foundation of China (71490732; YZ) and NIA/NIH (2P01AG031719, JWV, YZ).

*Danish longevity study and Danish longevity study II*

The Danish longevity study and the Danish longevity study II received funding from The National Program for Research Infrastructure 2007 (grant no. 09-063256), the Danish Agency for Science Technology and Innovation, the Velux Foundation, the US National Institute of Health (P01 AG08761), the Danish Agency for Science, Technology and Innovation/The Danish Council for Independent Research (grant no. 11-107308), **The Danish Interdisciplinary Research Council,** the European Union's Seventh Framework Programme (FP7/2007-2011) under grant agreement n° 259679, the INTERREG 4 A programme Syddanmark-Schleswig-K.E.R.N. (by EU funds from the European Regional Development Fund), **the CERA Foundation (Lyon), the AXA Research Fund, Paris, and The Health Foundation (Helsefonden), Copenhagen, Denmark.**

*Framingham Heart Study*

The FHS phenotype-genotype analyses were supported by the National Institute of Aging (R56AG29451). This research was conducted in part using data and resources from the Framingham Heart Study of the National Heart Lung and Blood Institute of the National Institutes of Health and Boston University School of Medicine. The analyses reflect intellectual input and resource development from the Framingham Heart Study investigators participating in the SNP Health Association Resource (SHARe) project. This work was partially supported by the National Heart, Lung and Blood Institute's Framingham Heart Study (Contract No. N01-HC-25195, HHSN268201500001) and its contract with Affymetrix, Inc for genotyping services (Contract No. N02-HL-6-4278). Douglas Kiel’s time was supported by a grant from the National Institute of Arthritis Musculoskeletal and Skin Diseases (R01 AR041398). The authors thank the participants for their dedication to the study. The authors are pleased to acknowledge that the computational work reported on in this paper was performed on the Shared Computing Cluster which is administered by Boston University’s Research Computing Services. URL: [www.bu.edu/tech/support/research/](http://www.bu.edu/tech/support/research/).

*Genetics of Healthy Aging Study*

The work described in this study was funded mainly by the EU GEHA Project contract no. LSHM-CT-2004-503-270. The work has additionally been supported by the following programs and agencies: The Competitive Research Funding of the Tampere University Hospital and Academy of Finland (Tampere); United States National Institute of Aging (PO1-AG08761) (Odense); The Innovation Oriented Research Program on Genomics (Senter-Novem IGE05007), the Centre for Medical Systems Biology (CMSB), and the National Institute for Healthy Ageing (NCHA 05060810), all in the framework of the Netherlands Genomics Initiative (NGI)/Netherlands Organisation for Scientific Research (NWO) (Leiden); The Institute for Ageing and Health and the UK NIHR Biomedical Research Centre for Ageing and Age-related disease award to the Newcastle-upon-Tyne Foundation Hospitals NHS Trust (Newcastle); Fondation Caisse d’Epargne Rh^one-Alpes Lyon CERAL (2004–2007) (Montpellier); Regione Autonoma della Sardegna (Sassari), European Union’s Seventh Framework Programme (FP7/2007-2011) IDEAL-ageing under grant agreement no. 259679.

*Genomics of extremely Overweight Young Adults*

The GOYA study was conducted as part of the activities of the Danish Obesity Research Centre (DanORC, www.danorc.dk) and The MRC centre for Causal Analyses in Translational Epidemiology (MRC CAiTE). The genotyping for GOYA was funded by the Wellcome Trust (WT 084762). GOYA is a nested study within The Danish National Birth Cohort which was established with major funding from the Danish National Research Foundation. Additional support for this cohort has been obtained from the Pharmacy Foundation, the Egmont Foundation, The March of Dimes Birth Defects Foundation, the Augustinus Foundation, and the Health Foundation.

*German longevity study*

The German longevity study was supported by the Cluster of Excellence ‘Inflammation at Interfaces’ (DFG - EXC 306). The biobank PopGen and the PopGen 2.0 network were supported by the BMBF (grant 01EY1103) and the federal state of Schleswig-Holstein.

*Health and Retirement Study*

HRS is supported by the National Institute on Aging (NIA U01AG009740). Genotyping was funded separately by NIA (RC2 AG036495, RC4 AG039029). Our genotyping was conducted by the NIH Center for Inherited Disease Research (CIDR) at Johns Hopkins University. Genotyping quality control and final preparation of the data were performed by the Genetics Coordinating Center at the University of Washington.

*Long Life Family Study*

We would like to acknowledge and thank the participants and staff of the Long Life Family Study. This work was supported by the National Institute on Aging (U01-AG023746, U01-AG023712, U01-AG023749, U01-AG023755, and U01-AG023744).

*Leiden Longevity Study*

The LLS has received funding from the European Union's Seventh Framework Programme (FP7/2007-2011) under grant agreement no 259679. This study was supported by a grant from the Innovation-Oriented Research Program on Genomics (SenterNovem IGE05007), the Centre for Medical Systems Biology, and the Netherlands Consortium for Healthy Ageing (grants 05040202 and 050060810), all in the framework of the Netherlands Genomics Initiative, Netherlands Organization for Scientific Research (NWO), Unilever Colworth, and by BBMRINL, a Research Infrastructure financed by the Dutch government (NWO 184.021.007). Joris Deelen is financially supported by the Alexander von Humboldt Foundation.

*Longevity Gene Project*

This work was supported by grants from NIH (R01AG044829, P01AG021654, R01AG046949, and RO1AG042188); the Nathan Shock Center of Excellence for the Biology of Aging (P30AG038072); and Glenn Center for the Biology of Human Aging Paul Glenn Foundation Grant.

*Osteoporotic Fractures in Men Study*

The Osteoporotic Fractures in Men (MrOS) Study is supported by National Institutes of Health funding. The following institutes provide support: the National Institute on Aging (NIA), the National Institute of Arthritis and Musculoskeletal and Skin Diseases (NIAMS), the National Center for Advancing Translational Sciences (NCATS), and NIH Roadmap for Medical Research under the following grant numbers: U01 AG027810, U01 AG042124, U01 AG042139, U01 AG042140, U01 AG042143, U01 AG042145, U01 AG042168, U01 AR066160, and UL1 TR000128. The National Institute of Arthritis and Musculoskeletal and Skin Diseases (NIAMS) provides funding for the MrOS ancillary study ‘Replication of candidate gene associations and bone strength phenotype in MrOS’ under the grant number R01 AR051124. The National Institute of Arthritis and Musculoskeletal and Skin Diseases (NIAMS) provides funding for the MrOS ancillary study ‘GWAS in MrOS and SOF’ under the grant number RC2 AR058973.

Computations were performed using the computer clusters and data storage resources of the HPCC, which were funded by grants from NSF (MRI-1429826) and NIH (1S10OD016290-01A1). Daniel S. Evans received funding from the NIA under grant number U24AG051129.

*Newcastle 85+ Study*

The Newcastle 85+ Study was supported by a combined grant from the Medical Research Council and Biotechnology and Biological Sciences Research Council (grant No G0500997), a grant from the Dunhill Medical Trust and a grant from the Newcastle Healthcare Charity. Support for further work with this cohort was provided by the Wellcome Trust (grants 087436/Z/08/Z and 102858/Z/13/Z) and the National Institute for Health Research (NIHR) Newcastle Biomedical Research Centre.

*Rotterdam study*

The generation and management of GWAS genotype data for the Rotterdam study was executed by the Human Genotyping Facility of the Genetic Laboratory of the Department of Internal Medicine, Erasmus MC, Rotterdam, The Netherlands. The GWAS datasets are supported by the Netherlands Organisation of Scientific Research NWO Investments (nr. 175.010.2005.011, 911-03-012), the Genetic Laboratory of the Department of Internal Medicine, Erasmus MC, the Research Institute for Diseases in the Elderly (014-93-015; RIDE2), the Netherlands Genomics Initiative (NGI)/Netherlands Organisation for Scientific Research (NWO) Netherlands Consortium for Healthy Aging (NCHA), project nr. 050-060-810. We thank Pascal Arp, Mila Jhamai, Marijn Verkerk, Lizbeth Herrera and Marjolein Peters, MSc, and Carolina Medina-Gomez, MSc, for their help in creating the GWAS database, and Karol Estrada, PhD, Yurii Aulchenko, PhD, and Carolina Medina-Gomez, MSc, for the creation and analysis of imputed data. The Rotterdam Study is funded by Erasmus Medical Center and Erasmus University, Rotterdam, Netherlands Organization for the Health Research and Development (ZonMw), the Research Institute for Diseases in the Elderly (RIDE), the Ministry of Education, Culture and Science, the Ministry for Health, Welfare and Sports, the European Commission (DG XII), the Municipality of Rotterdam, the H2020 Project Common mechanisms and pathways in Stroke and Alzheimer's disease (CoSTREAM) project (www.costream.eu, grant agreement No 667375), and the Alzheimer's Disease Apolipoprotein Pathology for Treatment Elucidation and Development consortium, which has received funding from the Innovative Medicines Initiative 2 Joint Undertaking. The authors are grateful to the study participants, the staff from the Rotterdam Study and the participating general practitioners and pharmacists.

*Study of Osteoporotic Fracture*

The Study of Osteoporotic Fractures (SOF) is supported by National Institutes of Health funding. The National Institute on Aging (NIA) provides support under the following grant numbers: R01 AG005407, R01 AR35582, R01 AR35583, R01 AR35584, R01 AG005394, R01 AG027574, and R01 AG027576.

*Supplementation in Vitamins and Mineral Antioxidants*

The SU.VI.MAX cohort team was funded by the French Institut National de la Santé et de la Recherche Médicale, the Institut National de la Recherche Agronomique, the Université Paris 13 and the Commissariat à L'Energie Atomique-Centre National de Génotypage.

*Vitality 90+*

The Vitality 90+ study has been financially supported by the Academy of Finland (grants 286284 (T.L), 132704 (M.H.), and 250602 (Coctel to M.J.); the Finnish Foundation of Cardiovascular Research (T.L.); the Tampere Tuberculosis Foundation (T.L, M.H.); the Emil Aaltonen Foundation (T.L); the Finnish Cultural Foundation, Pirkanmaa Regional fund (N.M); and the Yrjö Jahnsson Foundation (T.L., M.H.) This work was also supported by grants from the Competitive Research Fund of Pirkanmaa Hospital District (9M017,

9N013 to M.H, 9P002 to M.J. and X51001 for T.L.).

*Wellcome Trust Case Control Consortium 2*

This study makes use of data generated by the WTCCC2, funded by the Wellcome Trust under award 085475.

**Supplementary Figures**


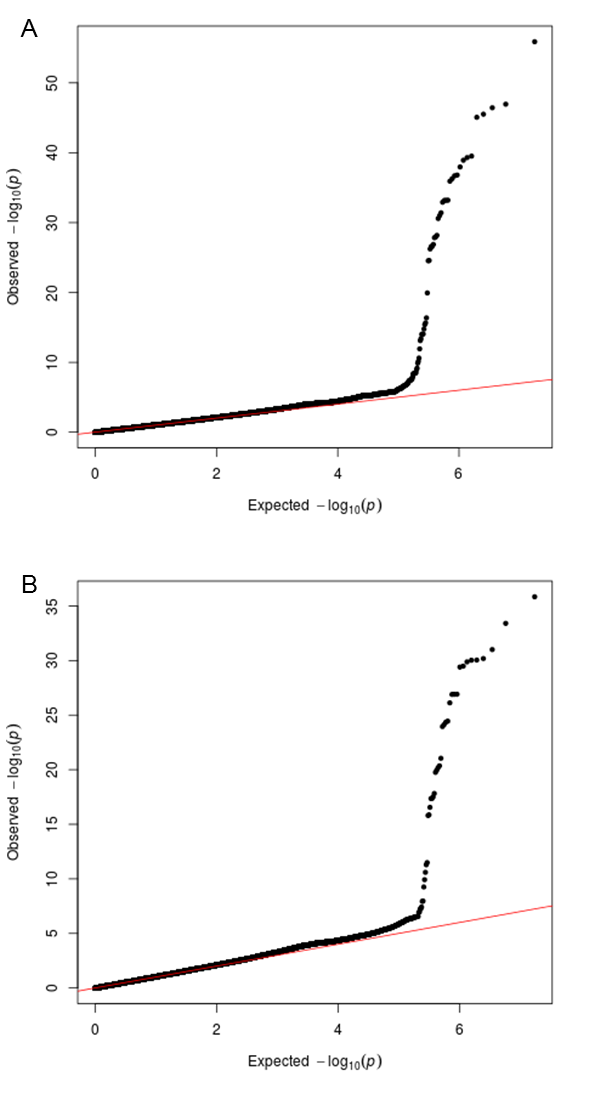


**Supplementary Figure 1. Quantile-quantile plots for the European genome-wide association meta-analyses**. Quantile-quantile plots of the expected versus (unadjusted) observed -log_10_ P-values for the European genome-wide association meta-analyses of the 90^th^ percentile cases versus all controls (λ = 1.036, a) and 99^th^ percentile cases versus all controls (λ = 1.036, b).


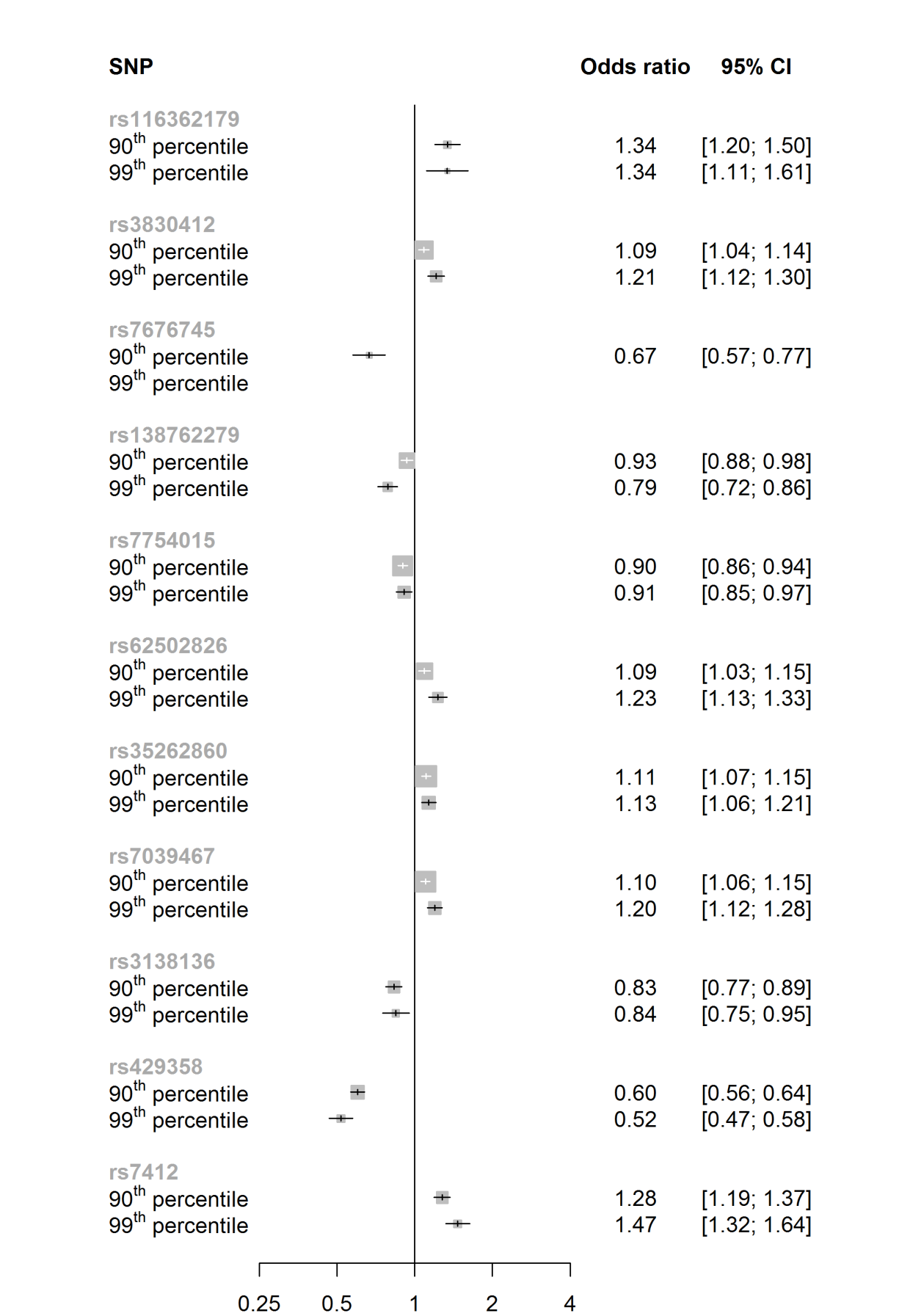


**Supplementary Figure 2. Results of the suggestive significant genetic variants from the European genome-wide association meta-analyses.** Forest plot for the suggestive significant genetic variants from the European genome-wide association meta-analyses of the 90^th^ and 99^th^ percentile versus all controls. We had insufficient studies with data for rs7676745 in the 99^th^ percentile versus all controls meta-analysis to reliable analyse this genetic variant due to its relatively low minor allele frequency.


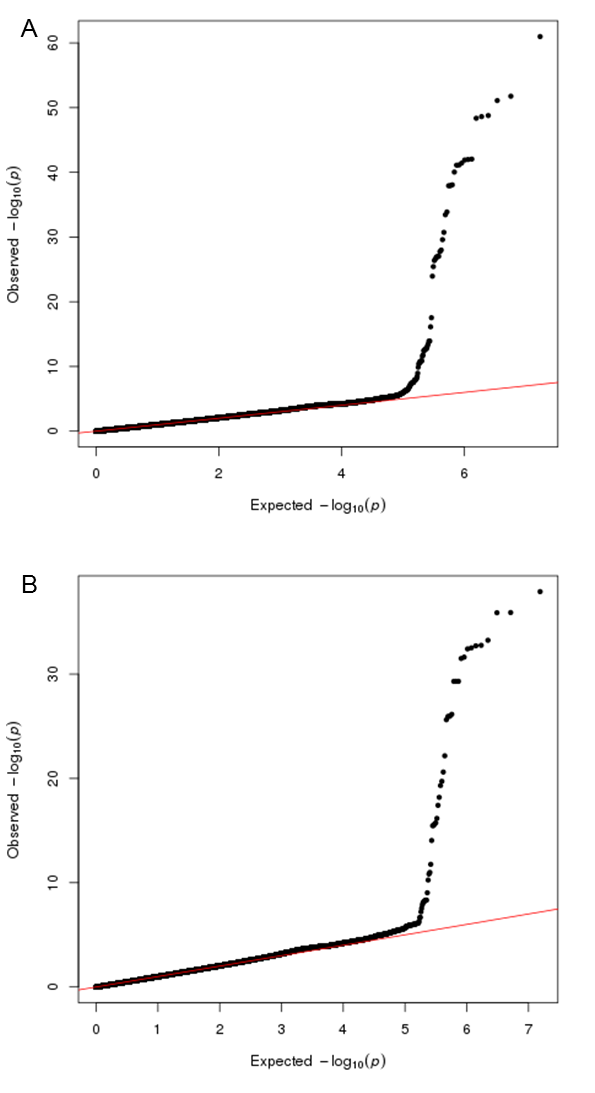


**Supplementary Figure 3. Quantile-quantile plots for the trans-ethnic genome-wide association meta-analyses**. Quantile-quantile plots of the expected versus (unadjusted) observed -log_10_ P-values for the trans-ethnic genome-wide association meta-analysis of the 90^th^ percentile cases versus all controls (λ = 0.97, a) and 99^th^ percentile cases versus all controls (λ = 0.93, b).

**
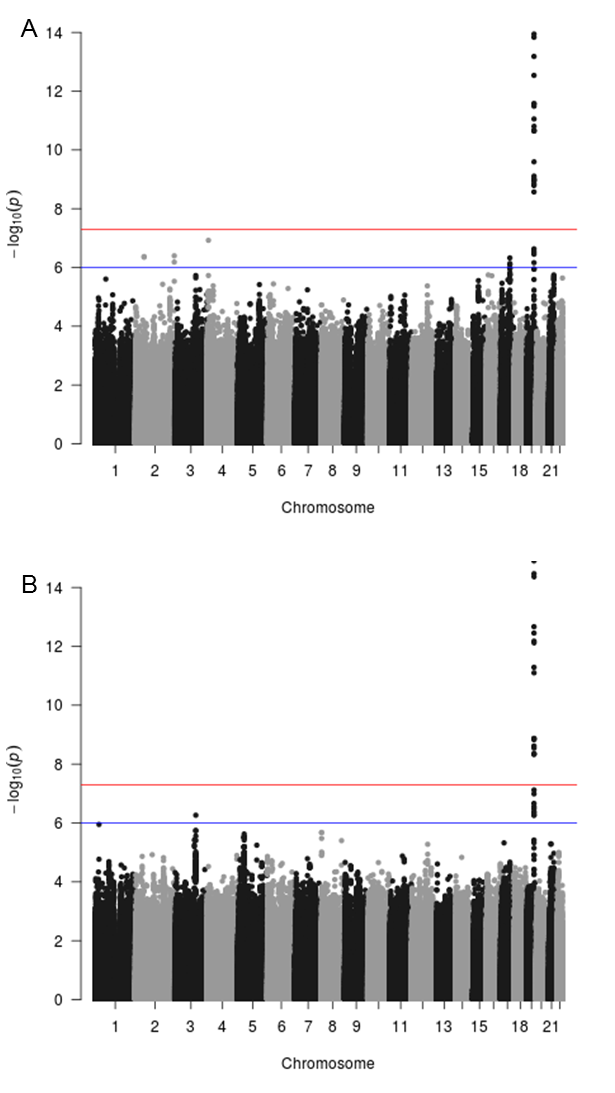
**

**Supplementary Figure 4. Results for the European genome-wide association meta-analyses using different control definitions.** Manhattan plot presenting the -log_10_ P-values from the European genome-wide association meta-analysis of the 90^th^ percentile cases versus all controls (a) or dead controls only (b). The red line indicates the threshold for genome-wide significance (P ≤5 x 10^-8^), while the blue line indicates the threshold for genetic variants that showed a suggestive significant association (P ≤1 x 10^-6^). For representation purposes, the maximum of the Y-axis was set to 14.


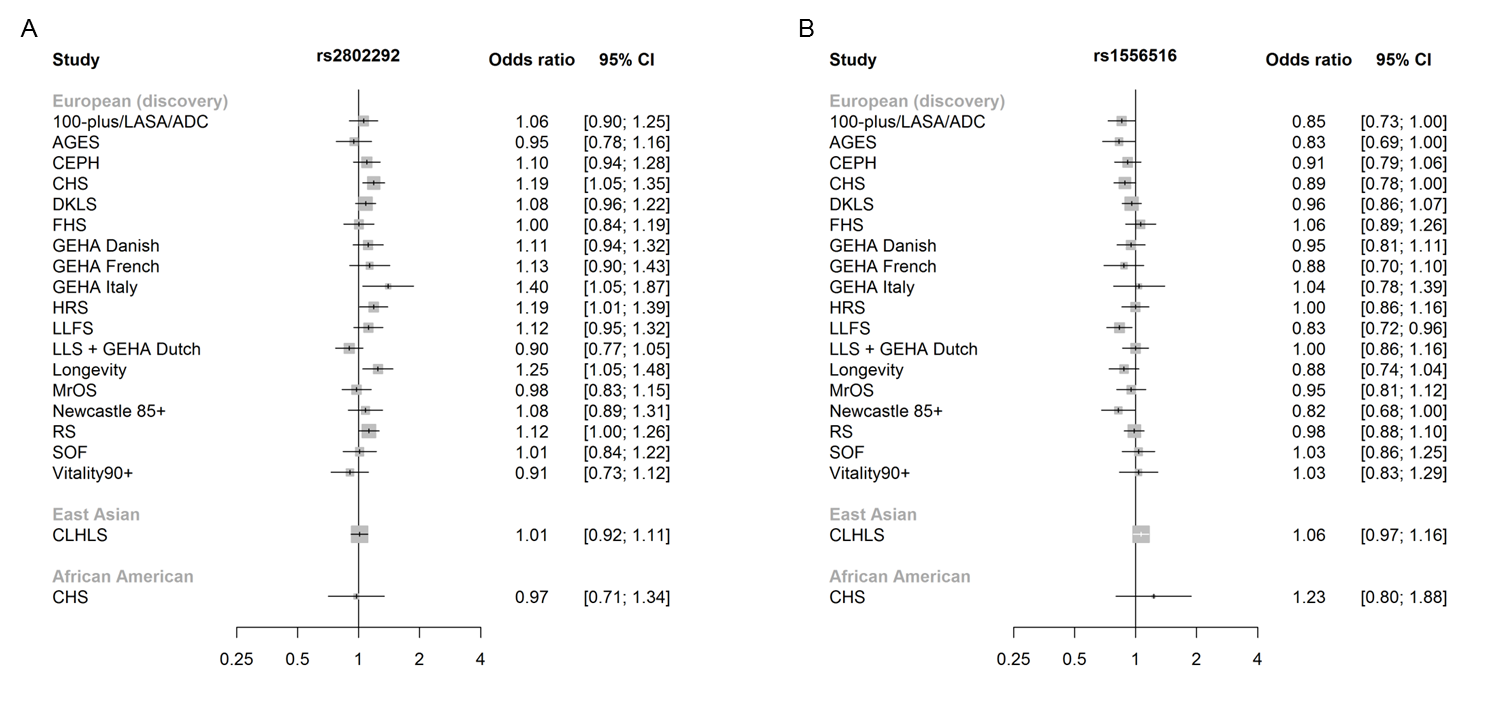
**Supplementary Figure 5. Study-specific results for the genetic variants in *FOXO3* and *CDKN2A/B*.** Forest plots for rs2802292 (a) and rs1556516 (b) based on the results from the 90^th^ percentile versus all controls analysis. The size of the boxes represents the sample size of the cohort.

**Supplementary Tables**

**Supplementary Table 1. Comparison of the results of the European genome-wide association meta-analyses of the 90^th^ percentile cases versus all controls and 90^th^ percentile cases versus dead controls.**

|  |  |  |  |  | **90^th^ percentile cases versus all controls** | | | | | | **90^th^ percentile cases versus dead controls** | | | | | |
| --- | --- | --- | --- | --- | --- | --- | --- | --- | --- | --- | --- | --- | --- | --- | --- | --- |
| **rsID** | **Chr** | **Position** | **Candidate/closest gene** | **Alleles**  **(EA/OA)** | **EAF** | **OR** | **95% CI** | **P** | **I^2^ (%)** | **P_het_** | **EAF** | **OR** | **95% CI** | **P** | **I^2^ (%)** | **P_het_** |
| rs7676745 | 4 | 8,565,547 | *GPR78* | A/G | 0.04 | 0.64 | 0.54 - 0.76 | 1.2 x 10^-7^ | 8.1 | 0.368 | 0.04 | 0.65 | 0.53 - 0.79 | 3.0 x 10^-5^ | 15.6 | 0.304 |
| rs7210199 | 17 | 55,933,994 | *CUEDC1* | T/C | 0.11 | 1.26 | 1.15 - 1.37 | 7.3 x 10^-7^ | 0.0 | 0.436 | 0.11 | 1.26 | 1.13 - 1.41 | 2.1 x 10^-5^ | 0.0 | 0.900 |
| rs429358 | 19 | 45,411,941 | *APOE* | C/T | 0.14 | 0.65 | 0.59 - 0.71 | 3.8 x 10^-22^ | 56.5 | 0.024 | 0.13 | 0.64 | 0.58 - 0.71 | 3.9 x 10^-19^ | 41.6 | 0.101 |

The *rsID* isbased on dbSNP build 150. The *Chr* and *Position* are based on Genome Reference Consortium Human Build 37 (GRCh37). *EA*, Effect allele; *OA*, Other allele; *EAF*, Effect allele frequency; *OR*, Odds ratio (i.e. odds to become long-lived when carrying the effect allele); *95% CI*, 95% confidence interval; *I^2^*, heterogeneity statistic; *P_het_*, P-value for heterogeneity.

**Supplementary Table 2. Results of previously identified loci for human lifespan in the generated UK Biobank parental longevity dataset.**

|  |  |  |  | **Timmers et al. 2019** | | | | **90^th^ percentile cases versus all controls** | | | |
| --- | --- | --- | --- | --- | --- | --- | --- | --- | --- | --- | --- |
| **rsID** | **Chr** | **Position** | **Candidate gene** | **EA** | **N** | **HR** | **P** | **EAF** | **OR** | **95% CI** | **P** |
| rs1230666 | 1 | 114,173,410 | *MAGI3* | G | 1,012,240 | 1.03 | 6.4 x 10^-9^ | 0.85 | 1.03 | 0.99 - 1.08 | 0.095 |
| rs1275922 | 2 | 26,932,887 | *KCNK3* | G | 1,012,240 | 1.03 | 6.0 x 10^-9^ | 0.75 | 1.06 | 1.03 - 1.10 | 1.9 x 10^-4^ |
| rs61348208 | 4 | 3,089,564 | *HTT* | T | 1,012,240 | 1.02 | 5.8 x 10^-9^ | 0.40 | 1.01 | 0.98 - 1.04 | 0.359 |
| rs34967069 | 6 | 32,591,248 | *HLA-DQA1* | T | 1,012,240 | 1.06 | 4.3 x 10^-9^ | 0.05 | 1.06 | 0.99 - 1.14 | 0.115 |
| rs10455872 | 6 | 161,010,118 | *LPA* | A | 1,012,240 | 1.08 | 8.5 x 10^-25^ | 0.92 | 1.08 | 1.03 - 1.14 | 0.003 |
| rs1556516 | 9 | 22,100,176 | *CDKN2B-AS1* | G | 1,012,240 | 1.03 | 7.5 x 10^-11^ | 0.50 | 1.04 | 1.01 - 1.07 | 0.003 |
| rs11065979 | 12 | 112,059,557 | *ATXN2/BRAP* | C | 1,012,240 | 1.03 | 1.0 x 10^-12^ | 0.56 | 1.07 | 1.04 - 1.10 | 4.5 x 10^-6^ |
| rs8042849 | 15 | 78,817,929 | *CHRNA3/5* | T | 1,012,240 | 1.04 | 1.6 x 10^-26^ | 0.66 | 1.06 | 1.03 - 1.09 | 1.0 x 10^-4^ |
| rs6224 | 15 | 91,423,543 | *FURIN/FES* | G | 1,012,240 | 1.03 | 1.3 x 10^-10^ | 0.53 | 1.05 | 1.03 - 1.08 | 2.4 x 10^-4^ |
| rs12924886 | 16 | 72,075,593 | *HP* | A | 1,012,240 | 1.03 | 1.4 x 10^-8^ | 0.81 | 1.02 | 0.99 - 1.06 | 0.180 |
| rs142158911 | 19 | 11,190,534 | *LDLR* | A | 1,012,240 | 1.04 | 8.1 x 10^-9^ | 0.12 | 1.05 | 1.00 - 1.10 | 0.034 |

The *rsID* is based on dbSNP build 150. The *Chr* and *Position* are based on Genome Reference Consortium Human Build 37 (GRCh37). *EA*, Effect allele; *HR*, Hazard ratio (i.e. hazard of death when carrying the effect allele); *EAF*, Effect allele frequency; *OR*, Odds ratio (i.e. odds to become long-lived when carrying the effect allele); *95% CI*, 95% confidence interval.

**Supplementary References**

1. Holstege H*, et al.* The 100-plus Study of cognitively healthy centenarians: rationale, design and cohort description. *Eur J Epidemiol* **33**, 1229-1249 (2018).

2. Huisman M*, et al.* Cohort profile: the Longitudinal Aging Study Amsterdam. *Int J Epidemiol* **40**, 868-876 (2011).

3. Hoogendijk EO*, et al.* The Longitudinal Aging Study Amsterdam: cohort update 2016 and major findings. *Eur J Epidemiol* **31**, 927-945 (2016).

4. van der Flier WM*, et al.* Optimizing patient care and research: the Amsterdam Dementia Cohort. *J Alzheimers Dis* **41**, 313-327 (2014).

5. van der Flier WM, Scheltens P. Amsterdam Dementia Cohort: Performing Research to Optimize Care. *J Alzheimers Dis* **62**, 1091-1111 (2018).

6. McCarthy S*, et al.* A reference panel of 64,976 haplotypes for genotype imputation. *Nat Genet* **48**, 1279-1283 (2016).

7. Das S*, et al.* Next-generation genotype imputation service and methods. *Nat Genet* **48**, 1284-1287 (2016).

8. O'Connell J*, et al.* A general approach for haplotype phasing across the full spectrum of relatedness. *PLoS Genet* **10**, e1004234 (2014).

9. Blanche H, Cabanne L, Sahbatou M, Thomas G. A study of French centenarians: are ACE and APOE associated with longevity? *C R Acad Sci III* **324**, 129-135 (2001).

10. Fried LP*, et al.* The Cardiovascular Health Study: design and rationale. *Ann Epidemiol* **1**, 263-276 (1991).

11. Raitakari OT*, et al.* Cohort profile: the cardiovascular risk in Young Finns Study. *Int J Epidemiol* **37**, 1220-1226 (2008).

12. Zeng Y. Towards Deeper Research and Better Policy for Healthy Aging --Using the Unique Data of Chinese Longitudinal Healthy Longevity Survey. *China Economic J* **5**, 131-149 (2012).

13. Zeng Y*, et al.* Novel loci and pathways significantly associated with longevity. *Sci Rep* **6**, 21243 (2016).

14. Bongaarts J. Book review on “Zeng Yi, D.L.Poston, D. A. Vlosky,and Danan Gu (eds.), Healthy Longevity in China: Demographic, Socioeconomic, and Psychological Dimensions”. *Population and Development Review* **35**, 452-453 (2009).

15. Goodkind D. Review on the book Healthy Longevity in China: Demographic, Socioeconomic, and Psychological Dimensions. *Population Studies* **63**, 1-7 (2009).

16. Rasmussen SH*, et al.* Cohort Profile: The 1895, 1905, 1910 and 1915 Danish Birth Cohort Studies - secular trends in the health and functioning of the very old. *Int J Epidemiol* **46**, 1746-1746j (2017).

17. Robine JM, Cheung SL, Saito Y, Jeune B, Parker MG, Herrmann FR. Centenarians Today: New Insights on Selection from the 5-COOP Study. *Curr Gerontol Geriatr Res* **2010**, 120354 (2010).

18. Skytthe A, Kyvik K, Holm NV, Vaupel JW, Christensen K. The Danish Twin Registry: 127 birth cohorts of twins. *Twin Res* **5**, 352-357 (2002).

19. Miller SA, Dykes DD, Polesky HF. A simple salting out procedure for extracting DNA from human nucleated cells. *Nucleic Acids Res* **16**, 1215 (1988).

20. Skytthe A*, et al.* The Danish Twin Registry: linking surveys, national registers, and biological information. *Twin Res Hum Genet* **16**, 104-111 (2013).

21. Dawber TR, Kannel WB. The Framingham study. An epidemiological approach to coronary heart disease. *Circulation* **34**, 553-555 (1966).

22. Feinleib M, Kannel WB, Garrison RJ, McNamara PM, Castelli WP. The Framingham Offspring Study. Design and preliminary data. *Prev Med* **4**, 518-525 (1975).

23. Splansky GL*, et al.* The Third Generation Cohort of the National Heart, Lung, and Blood Institute's Framingham Heart Study: design, recruitment, and initial examination. *Am J Epidemiol* **165**, 1328-1335 (2007).

24. Skytthe A*, et al.* Design, recruitment, logistics, and data management of the GEHA (Genetics of Healthy Ageing) project. *Exp Gerontol* **46**, 934-945 (2011).

25. Paternoster L*, et al.* Genome-wide population-based association study of extremely overweight young adults--the GOYA study. *PLoS One* **6**, e24303 (2011).

26. Flachsbart F*, et al.* Immunochip analysis identifies association of the RAD50/IL13 region with human longevity. *Aging Cell* **15**, 585-588 (2016).

27. Flachsbart F*, et al.* Identification and characterization of two functional variants in the human longevity gene FOXO3. *Nat Commun* **8**, 2063 (2017).

28. Chang CC, Chow CC, Tellier LC, Vattikuti S, Purcell SM, Lee JJ. Second-generation PLINK: rising to the challenge of larger and richer datasets. *Gigascience* **4**, 7 (2015).

29. Sonnega A, Faul JD, Ofstedal MB, Langa KM, Phillips JW, Weir DR. Cohort Profile: the Health and Retirement Study (HRS). *Int J Epidemiol* **43**, 576-585 (2014).

30. Sebastiani P*, et al.* A family longevity selection score: ranking sibships by their longevity, size, and availability for study. *Am J Epidemiol* **170**, 1555-1562 (2009).

31. Newman AB*, et al.* Health and function of participants in the Long Life Family Study: A comparison with other cohorts. *Aging (Albany NY)* **3**, 63-76 (2011).

32. Elo IT, Mykyta L, Sebastiani P, Christensen K, Glynn NW, Perls T. Age validation in the long life family study through a linkage to early-life census records. *J Gerontol B Psychol Sci Soc Sci* **68**, 580-585 (2013).

33. Bae HT*, et al.* Genome-wide association study of personality traits in the long life family study. *Front Genet* **4**, 65 (2013).

34. Schoenmaker M*, et al.* Evidence of genetic enrichment for exceptional survival using a family approach: the Leiden Longevity Study. *Eur J Hum Genet* **14**, 79-84 (2006).

35. Beekman M, Blauw GJ, Houwing-Duistermaat JJ, Brandt BW, Westendorp RG, Slagboom PE. Chromosome 4q25, microsomal transfer protein gene, and human longevity: novel data and a meta-analysis of association studies. *J Gerontol A Biol Sci Med Sci* **61**, 355-362 (2006).

36. Deelen J*, et al.* Genome-wide association meta-analysis of human longevity identifies a novel locus conferring survival beyond 90 years of age. *Hum Mol Genet* **23**, 4420-4432 (2014).

37. Barzilai N*, et al.* Unique lipoprotein phenotype and genotype associated with exceptional longevity. *JAMA* **290**, 2030-2040 (2003).

38. Huffman DM*, et al.* Distinguishing between longevity and buffered-deleterious genotypes for exceptional human longevity: the case of the MTP gene. *J Gerontol A Biol Sci Med Sci* **67**, 1153-1160 (2012).

39. Eny KM*, et al.* GWAS identifies an NAT2 acetylator status tag single nucleotide polymorphism to be a major locus for skin fluorescence. *Diabetologia* **57**, 1623-1634 (2014).

40. Collerton J*, et al.* Health and disease in 85 year olds: baseline findings from the Newcastle 85+ cohort study. *BMJ* **339**, b4904 (2009).

41. Ikram MA*, et al.* The Rotterdam Study: 2018 update on objectives, design and main results. *Eur J Epidemiol* **32**, 807-850 (2017).

42. Hercberg S*, et al.* Background and rationale behind the SU.VI.MAX Study, a prevention trial using nutritional doses of a combination of antioxidant vitamins and minerals to reduce cardiovascular diseases and cancers. SUpplementation en VItamines et Mineraux AntioXydants Study. *Int J Vitam Nutr Res* **68**, 3-20 (1998).

43. Lehtimaki T*, et al.* Interleukin-6 modulates plasma cholesterol and C-reactive protein concentrations in nonagenarians. *J Am Geriatr Soc* **53**, 1552-1558 (2005).

44. Cordell HJ*, et al.* Genome-wide association study of multiple congenital heart disease phenotypes identifies a susceptibility locus for atrial septal defect at chromosome 4p16. *Nat Genet* **45**, 822-824 (2013).
