# Supplementary material for: A meta-analysis of genome-wide association studies identifies multiple longevity genes
Source: Nat Commun. 2019 Aug 14;10:3669. doi: 10.1038/s41467-019-11558-2 (PMC6694136; doi:10.1038/s41467-019-11558-2)
Supplement: Supplementary file 8 — Description of Additional Supplementary Files [file 41467_2019_11558_MOESM8_ESM.docx]

**Title: Supplementary Data 1**
**Description:** Results of previously identified loci for human lifespan and longevity in the European genome-wide association meta-analyses of the 90^th^ percentile cases versus dead controls and 99^th^ percentile cases versus all controls.

**Title: Supplementary Data 2**
**Description:** Results from MetaXcan and colocalization of GWAS and eQTL results.

**Title: Supplementary Data 3**
**Description:** Full results of the genetic correlation analyses of the 90^th^ and 99^th^ percentile phenotypes with other diseases and traits.

**Title: Supplementary Data 4**
**Description:** Demographics of the cohorts included in the European and /or trans-ethnic genome-wide association meta-analyses.

**Title: Supplementary Data 5**
**Description:** Details of the genotyping, quality control and imputation of the cohorts included in the European and /or trans-ethnic genome-wide association meta-analyses.
