# Supplementary material for: A meta-analysis of genome-wide association studies identifies multiple longevity genes
Source: Nat Commun. 2019 Aug 14;10:3669. doi: 10.1038/s41467-019-11558-2 (PMC6694136; doi:10.1038/s41467-019-11558-2)
Supplement: Supplementary file 9 — Reporting Summary [file 41467_2019_11558_MOESM9_ESM.pdf]

nature research

Corresponding author(s): Joris Deelen, Daniel S. Evans, P. Eline Slagboom and Joanne M. Murabito

Last updated by author(s): Jul 16, 2019

Reporting Summary

Nature Research wishes to improve the reproducibility of the work that we publish. This form provides structure for consistency and transparency in reporting. For further information on Nature Research policies, see Authors & Referees and the Editorial Policy Checklist.

Statistics

For all statistical analyses, confirm that the following items are present in the figure legend, table legend, main text, or Methods section.

n/a	Confirmed	
☐	☒	The exact sample size (*n*) for each experimental group/condition, given as a discrete number and unit of measurement
☐	☒	A statement on whether measurements were taken from distinct samples or whether the same sample was measured repeatedly
☐	☒	The statistical test(s) used AND whether they are one- or two-sided<br>*Only common tests should be described solely by name; describe more complex techniques in the Methods section.*
☐	☒	A description of all covariates tested
☐	☒	A description of any assumptions or corrections, such as tests of normality and adjustment for multiple comparisons
☐	☒	A full description of the statistical parameters including central tendency (e.g. means) or other basic estimates (e.g. regression coefficient) AND variation (e.g. standard deviation) or associated estimates of uncertainty (e.g. confidence intervals)
☒	☐	For null hypothesis testing, the test statistic (e.g. *F*, *t*, *r*) with confidence intervals, effect sizes, degrees of freedom and *P* value noted<br>*Give P values as exact values whenever suitable.*
☒	☐	For Bayesian analysis, information on the choice of priors and Markov chain Monte Carlo settings
☒	☐	For hierarchical and complex designs, identification of the appropriate level for tests and full reporting of outcomes
☒	☐	Estimates of effect sizes (e.g. Cohen's *d*, Pearson's *r*), indicating how they were calculated

Our web collection on statistics for biologists contains articles on many of the points above.

Software and code

Policy information about availability of computer code

Data collection	Not applicable
Data analysis	Data analyses were performed using publicly available software. The links and references are provided in the manuscript. In short, we used the following programs:<br><br>Imputation; SHAPEIT, IMPUTE, MaCH, minimac<br>Association analyses of individuals datasets; SNPTEST, PLINK, ProbABEL, R, SAS, CC-assoc<br>Quality control of individuals datasets; EasyQC<br>Meta-analyses; METAL and METASOFT<br>Conditional analyses; SNPTEST<br>Gene-level association analysis; MetaXcan<br>Genetic correlation analysis; LD score regression (including LDHub)<br>Power calculation; Genetic Association Study Power Calculator

For manuscripts utilizing custom algorithms or software that are central to the research but not yet described in published literature, software must be made available to editors/reviewers. We strongly encourage code deposition in a community repository (e.g. GitHub). See the Nature Research guidelines for submitting code & software for further information.

Data

Policy information about availability of data

All manuscripts must include a data availability statement. This statement should provide the following information, where applicable:

- Accession codes, unique identifiers, or web links for publicly available datasets
- A list of figures that have associated raw data
- A description of any restrictions on data availability

The full meta-analyses summary statistics are available for download at www.longevitygenomics.org/downloads and through GRASP (https://grasp.nhlbi.nih.gov/FullResults.aspx). All other data that supports the findings of this study are available from the corresponding authors upon request.

Field-specific reporting

Please select the one below that is the best fit for your research. If you are not sure, read the appropriate sections before making your selection.

☒ Life sciences ☐ Behavioural & social sciences ☐ Ecological, evolutionary & environmental sciences

For a reference copy of the document with all sections, see nature.com/documents/nr-reporting-summary-flat.pdf

Life sciences study design

All studies must disclose on these points even when the disclosure is negative.

Sample size	The sample size of the study was based on the number of samples that met our case/control-definition and had genome-wide genotyping data available that passed quality control.
Data exclusions	Samples were excluded based on call rate. Moreover, we excluded ethnic outliers, duplicates, related individuals, gender mismatches, family mismatches, mismatches with previous genotyping, and samples with high levels of heterozygosity. Genetic variants were excluded based on the minor allele frequency, call rate, Hardy-Weinberg equilibrium P-value, imputation quality, and expected minor allele count.
Replication	Replication was performed in the two cohorts in which de novo genotyping, using predesigned Taqman SNP Genotyping Assays, was applied. Moreover, we tried to validate our findings in two newly created UK Biobank parental longevity datasets and the parental lifespan dataset created by Timmers and colleagues.
Randomization	Not applicable
Blinding	Not applicable

Reporting for specific materials, systems and methods

We require information from authors about some types of materials, experimental systems and methods used in many studies. Here, indicate whether each material, system or method listed is relevant to your study. If you are not sure if a list item applies to your research, read the appropriate section before selecting a response.

Materials & experimental systems

n/a	Involved in the study	
☒	☐	Antibodies
☒	☐	Eukaryotic cell lines
☒	☐	Palaeontology
☒	☐	Animals and other organisms
☐	☒	Human research participants
☒	☐	Clinical data

Methods

n/a	Involved in the study	
☒	☐	ChIP-seq
☒	☐	Flow cytometry
☒	☐	MRI-based neuroimaging

Human research participants

Policy information about studies involving human research participants

Population characteristics	We used individuals from populations of European, Asian, or African-American descent. More details on the characteristics of the cohorts included in this manuscript are provided in the Supplementary information.
Recruitment	Details on the recruitment procedures for the cohorts included in this manuscript are provided in the Supplementary information.

Ethics oversight | We have complied with all relevant ethical regulations for work with human subjects. All participants provided written informed consent and the studies were approved by the relevant institutional review boards.

Note that full information on the approval of the study protocol must also be provided in the manuscript.
